# RETRACTED: Sá et al. Involvement of GPR43 Receptor in Effect of *Lacticaseibacillus rhamnosus* on Murine Steroid Resistant Chronic Obstructive Pulmonary Disease: Relevance to Pro-Inflammatory Mediators and Oxidative Stress in Human Macrophages. *Nutrients* 2024, *16*, 1509

**DOI:** 10.3390/nu17152513

**Published:** 2025-07-31

**Authors:** Ana Karolina Sá, Fabiana Olímpio, Jessica Vasconcelos, Paloma Rosa, Hugo Caire Faria Neto, Carlos Rocha, Maurício Frota Camacho, Uilla Barcick, Andre Zelanis, Flavio Aimbire

**Affiliations:** 1 Department of Medicine, Postgraduate Program in Translational Medicine, Federal University of São Paulo (UNIFESP), Rua Pedro De Toledo 720–2 Andar, Vila Clementino, São Paulo 04039-002, Brazil; ana.karolina25@unifesp.br (A.K.S.); fabiana.olimpio@unifesp.br (F.O.); jessica.carvalho07@unifesp.br (J.V.); cristina.paloma@unifesp.br (P.R.); 2 Laboratory of Immunopharmacology, Institute of Science and Technology, Federal University of São Paulo, Rua Talim, 330, Vila Nair, São José dos Campos 12231-280, Brazil; 3 Laboratory of Immunopharmacology, Oswaldo Cruz Foundation Fundação Oswaldo Cruz, Av. Brazil, Rio de Janeiro 4036, Brazil; hugo.caire@fiocruz.br; 4 Medical School, Group of Phytocomplexes and Cell Signaling, Anhembi Morumbi University, São José dos Campos 04039-002, Brazil; carlosrocha.hd@gmail.com; 5 Functional Proteomics Laboratory, Institute of Science and Technology, Federal University of São Paulo, São José dos Campos 12231-280, Brazil; mauriciof.camacho@gmail.com (M.F.C.); lila.barcick@gmail.com (U.B.); andre.zelanis@unifesp.br (A.Z.); 6 Postgraduate Program in Pharmaceutical Sciences, Evangelical University of Goiás (UniEvangélica), Avenida Universitária Km 3,5, Anápolis 75083-515, Brazil

The journal retracts the article titled “Involvement of GPR43 Receptor in Effect of *Lacticaseibacillus rhamnosus* on Murine Steroid Resistant Chronic Obstructive Pulmonary Disease: Relevance to Pro-Inflammatory Mediators and Oxidative Stress in Human Macrophages” [1], cited above.

Following publication, the authors raised concerns about inaccuracies in the findings presented in this study [1] and requested an amendment for the removal of 2 parts of Section 11; “Histology and Morphometric Analysis”, and Table 4 “Effect of Dexamethasone on Airway Remodeling in COPD Mice”, as well as Figures 2 and 5.

Following an evaluation of the author’s request, the Editorial Board determined that the extent of the requested changes would affect the validity of the remaining data and significantly affect the study’s conclusions. As a result, the Editorial Board decided to retract this article [1], as per MDPI’s retraction policy (https://www.mdpi.com/ethics#_bookmark30).

This retraction was approved by the Editors in Chief of the journal *Nutrients*.

The authors agree to this retraction and wish to apologize to the readership for any inconvenience caused.
